# Knowledge, Perceptions and Attitudes toward Chronic Pain and Its Management: A Cross-Sectional Survey of Frontline Pharmacists in Ontario, Canada

**DOI:** 10.1371/journal.pone.0157151

**Published:** 2016-06-07

**Authors:** Tejal Patel, Feng Chang, Heba Tallah Mohammed, Lalitha Raman-Wilms, Jane Jurcic, Ayesha Khan, Beth Sproule

**Affiliations:** 1 School of Pharmacy, University of Waterloo, Kitchener, Ontario, Canada; 2 Centre for Family Medicine Family Health Team, Kitchener, Ontario, Canada; 3 Department of Family Medicine, DeGroote School of Medicine, McMaster University, Hamilton, Ontario, Canada; 4 Gateway Centre of Excellence in Rural Health, Seaforth, Ontario, Canada; 5 Department of Community Medicine, Aim Shams University, Cairo, Egypt; 6 Leslie Dan Faculty of Pharmacy, University of Toronto, Toronto, Ontario, Canada; 7 Centre for Addiction and Mental Health, Toronto, Ontario, Canada; Sudbury Regional Hospital, CANADA

## Abstract

The treatment of chronic pain consumes a significant share of primary care. Community and family health team pharmacists frequently see patients with chronic pain, thus have the opportunity to improve their care. To assess the knowledge, perceptions, and attitudes of Ontario pharmacists, we invited 5,324 Ontario pharmacists, to participate in an online survey we developed using Qualtrics. The 31-question survey gathered demographic information, assessed pharmacists’ knowledge of three chronic pain conditions; chronic lower back pain (CLBP, eight true/false statements); chronic headache disorder (CHD, eight true/false statements) and painful diabetic neuropathy (PDN, seven true/false statements), and their attitudes toward and perceptions of patients with these conditions, and knowledge, attitudes, and perceptions of opioids in pain management. We received 688 responses (12.9%) and 392 pharmacists completed the survey. The mean age of respondents was 48.5 years and 48.5% were male. More than 50% of respondents were in practice for more than 20 years and 58.7% worked 25–40 hours per week. The mean knowledge scores were 4.5/8, 5.5/8, and 5.3/8 for CBLP, CHD, and PDN respectively. While 95% of respondents were aware of the increasing death rates due to opioid use, only half were familiar with the Canadian guideline for safe opioid prescribing for non-cancer use. Responses were compared based on gender, time in practice and location of practice. Pharmacists with more than ten years of experience scored significantly higher than those with less experience. Fewer differences were found in comparisons of gender and location of practice. Safe and effective care of chronic pain patients, particularly with opioids, will require additional pharmacist education.

## Introduction

Chronic pain is a common condition affecting all age groups, ethnicities, and both sexes. It has significant impact on the quality of life for the individual, and affects their family, their employer, and society at large. Estimates of the prevalence of chronic pain vary considerably across the world. While a Canadian study conducted in 2011 found that 15% to 19% of adults 20 years of age and older reported living with chronic pain [1], other studies have determined that the prevalence of persistent pain in this demographic ranges from 5% to 55% [2–4]. Chronic pain places a significant economic burden on patients and society, with related healthcare costs in Canada estimated at more than CDN $6 billion and losses attributable to decreased work productivity and sick days estimated at CDN $37 billion per year [5,6]. For many people, chronic pain reduces their day-to-day functioning and lowers their quality of life, specifically with respect to sleep, cognitive function, mood, and mental health [2,7–9].

In primary care, an average of one out of every five persons report persistent pain [2]. In Europe, 70% of patients reported that their pain was managed by their general practitioner, 27% by their orthopedic specialist and only 2% reported being treated by a pain management specialist [8]. Canadian primary care physicians report a mean of 58 (range 5–300, proportion not provided) patients presenting for the management of moderate to severe pain per month, of which 85% were being treated for chronic non-cancer pain [10]. Effective pain management often requires multiple modalities and typically, is best managed within a collaborative health professional team [11,12]. Although medications are a key component of pain management, pharmacotherapy can present challenges to patient care. Such treatment may be ineffective or marginally effective [8], cause side effects leading to discontinuation [8], and be overly reliant on chronic opioids including non-prescription codeine products [10,13].

The increase in prescription opioid abuse over the past decade is of particular concern [14,15]. Rates of opioid misuse in patients with chronic pain have been estimated at 21% to 29% and rates of addiction from 8% to 12% [16]. The relationship between inadvertent overdose deaths and daily opioid doses over 200 mg morphine equivalents [17] or over 100 mg morphine equivalents [18] has been established [17,18], adding to the complexity and urgency of improving clinical management of chronic pain with opioids.

Given that pharmacists are medication experts, are among the most accessible healthcare professionals, and have frequent contact with patients living with chronic pain [19], those that practice in the community are in an ideal position to improve assessment, treatment, and management of chronic pain [20]. In a Canadian survey of 137 family physicians and 129 community pharmacists in Quebec, the mean scores on a questionnaire designed to investigate the knowledge, attitudes, and beliefs related to chronic non-cancer pain were 62.5% for pharmacists and 69.4% for physicians. Additionally, the questionnaire was designed to investigate initial pain assessments, implementation of treatment plans, management of longitudinal care, and environmental issues with a limited number of questions addressing knowledge of chronic non-cancer pain [21]. Such a study has not been conducted among community pharmacists in Ontario.

Therefore, the overarching aim of this project was to investigate the knowledge, attitudes, perceptions, and self-reported efficacy among pharmacists as related to the management of chronic non-cancer pain, with the intent to subsequently develop a specialized training program for community pharmacists to manage chronic pain more effectively while improving the safety of opioid therapy. We defined chronic pain as pain lasting for more than three months and we focused specifically on three highly prevalent pain conditions: chronic low back pain (CLBP), chronic headache disorders (CHD), and painful diabetic neuropathy (PDN) [22–24]. We report the development, administration, and evaluation of a survey to assess the knowledge, perceptions, and attitudes of primary care pharmacists in Ontario, Canada with respect to these chronic pain conditions.

## Methods

### Study design and population

The study, approved by the Institutional Review Boards at the University of Waterloo and the University of Toronto, is a cross-sectional survey of pharmacists providing primary care to patients in Ontario, Canada at community pharmacies, community health centers, and family health teams. The study population consisted of pharmacists registered in part A of the Register of Pharmacists with the Ontario College of Pharmacists (OCP), the province’s regulatory body. Part A pharmacists are those that provide active patient care, dispense, sell, and compound medications, and supervise a pharmacy where medications are kept (OCP website, Pharmacy Act). Of the 13,072 Part A pharmacists in Ontario, 5,324 expressed an interest in participating in research during their initial registration with the OCP or when renewing their annual license. The OCP provided a list of names and email addresses of these pharmacists to the Ontario Pharmacy Research Collaboration, a multidisciplinary pharmacy practice research program, of which the authors of this study are members.

All 5,324 pharmacists in OCP’s registry were invited to participate in an online survey by email. The initial invitation and a description of the study’s objectives were sent on September 15, 2014. Two additional reminders were sent by email on September 29, 2014 and October 13, 2014. Pharmacists were given the opportunity complete the survey on paper and return it by fax or mail, but none had used this option. The link to the survey was active for 6 weeks and directed pharmacists to Qualtrics, an online survey administration tool (Qualtrics, Provo, UT, September, 2014). Respondents were anonymous; i.e., responses could not be linked to responder. At the end of the survey, respondents were provided another link to enter their name and contact details in a draw for an iPad Air in appreciation for their time, along with an opportunity to indicate interest in participating in future educational opportunities; however, this information was not linked to survey respondents.

### Survey instrument

Previously published literature examining knowledge, attitudes, and perceptions toward chronic pain were reviewed [25–41], from which relevant survey questions were used, adapted, or modified with permission to create a survey instrument that addressed the goals of this study. The survey assessed pharmacists’ knowledge of CLBP, CHDs, and PDN, their attitudes toward and perceptions of patients with these conditions, and knowledge, attitudes, and perceptions of opioids in pain management. The survey instrument included questions on practice patterns and self-efficacy.

The developed survey was pilot tested by five community and primary care pharmacists to assess wording, structure, layout, and readability, and to provide an estimate for completion time. The survey was modified based on their feedback. The instrument’s face and content validity were determined through multiple separate reviews and discussions by the study’s authors. It was estimated the survey would take 20 minutes to complete.

The final survey instrument consisted of 31 questions, several of which had sub-questions. Of these questions, seven assessed pharmacists’ knowledge, five investigated attitudes and perceptions, and seven captured demographic characteristics. An additional twelve questions addressed pharmacy practice patterns, and pharmacists’ self-efficacy in managing chronic pain, but these data are not presented in this paper.

Within the knowledge-assessment questions, were eight statements on CLBP, eight on CHDs and seven on PDN that responders were asked to indicate as true, false, or not sure. The other four knowledge questions were on opioid use as follows: one yes/no question asking respondents if they were familiar with the Canadian guideline for the safe and effective use of opioids in chronic non-cancer pain, one question where respondents were asked to provide the guideline’s recommended “watchful dose” of opioids per day in mg morphine equivalents, and two true/false statements assessing knowledge about the rise of overdoses related to prescription opioids in Ontario and the potential for patients with a legitimate pain problem to become addicted to prescription opioids.

In two of the five questions addressing pharmacists’ attitudes and perceptions of chronic pain, participants were asked to respond to questions on the importance of incorporating complementary treatment and importance of opioids in the treatment of CLBP, CHDs, and PDN on a five-point Likert scale (options as follows: always, very often, sometimes, rarely, never). The remaining three attitudes and perceptions questions asked participants to indicate how often under-dosing was the reason for ongoing pain for each condition on a five-point Likert scale (options as follows: always, very often, sometimes, rarely, never), how much improvement was expected during the course of illness for each condition on a five-point Likert scale (options as follows: full improvement, major improvement, moderate improvement, little improvement, no improvement), and how they felt when approached by patients with CLBP, CHDs and PDN, each on a three-point Likert scale (options as follows: burdened, neutral, comfortable). Respondents were permitted to answer as many questions as they desired and to skip over questions.

### Data analysis

Study data were recorded using Qualtrics (www.qualtrics.com; Qualtrics, Provo, UT, September 2014) and analyzed using the Statistical Package for Social Sciences (SPSS; IBM Corp, Armonk, NY. Version 22; 2013). Descriptive statistics of survey respondents and survey results were calculated. Chi-square tests were used to examine the relationship between demographic variables (gender, years of practice, hours of practice, and population size) and pharmacists’ attitudes, knowledge, and perceptions toward treatment of chronic pain. Odds ratios and confidence intervals were estimated for statistically significant variables.

A post-hoc calculation for sample size indicated a sample size of 385 was required to detect significant differences for a margin of error of 0.05 and an assumed standard deviation of 0.5.

## Results

### Response rate

Of the 5,324 pharmacists asked to take the online survey, 52 participant email addresses were undeliverable. A further 95 respondents indicated they did not practice in either a community pharmacy or a family health team/community health center, thus were automatically excluded from the survey. A total of 668 (12.9%) pharmacists responded to the survey, of whom 392 completed the survey.

### Demographic characteristics

The mean age of responding pharmacists was 48.5 years (SD 11.5 years), 51.5% were female, 51.3% had been in practice for more than 20 years, and 80.6% worked at least 25 hours a week (Table 1). As summarized in Table 1, 12% of respondents had a rural practice (population <10,000), 24.2% small urban (10,000–100,000), 18.1% medium urban (100,001–250,000), 27.0% large urban (250,001–750,000), and 18.6% metropolitan (>750,000).

**Table 1:** Demographic characteristics of respondents.

	Variables	n (%)
**Gender**	Male	190 (48.5%)
	Female	202 (51.5%)
	Total	392
Education*	BSc in pharmacy	370 (94.4%)
	Entry-level PharmD	4 (1.0%)
	PharmD	16 (4.1%)
	MScPhm	16 (4.1%)
	Residency trained	24 (6.1%)
	Fellowship trained	0
**Years of Practice**	≤5 years	52 (13.3%)
	5–10 years	55 (14.0%)
	11–15 years	56 (14.3%)
	16–20 years	28 (7.1%)
	>20 years	201 (51.3%)
	Total	392
**Hours of practice**	<6 hours	15 (3.8%)
	8–24 hours	61 (15.6%)
	25–40 hours	230 (58.7%)
	>41 hours	86 (21.9%)
	Total	392
**Population size**	Rural	47 (12.0%)
	Small urban	95 (24.2%)
	Medium urban	71 (18.1%)
	Large urban	106 (27.0%)
	Metropolitan	73 (18.6%)
	Total	392

* Total does not add up to 392 as participants were invited to choose all that applied in terms of education and training.

### Knowledge, perceptions and attitudes

#### Knowledge of CLBP, CHDs, and PDN

Pharmacists’ knowledge of the pathophysiology and treatment of CLBP, CHDs and PDN was assessed by 23 true/false statements: eight on CLBP, eight on CHDs, and seven on PDN (Table 2). The mean correct score for all questions was 15.28/23 (66%; range from 0–96%). The mean correct scores for the CLBP, CHDs and PDN components were 4.49/8 (55%), 5.49/8 (69%) and 5.34/7 (76%), respectively.

**Table 2:** Proportion of correct and incorrect answers to knowledge questions on Chronic Low Back Pain (CLBP), Chronic Headache Disorders (CHDs) and Peripheral Diabetic neuropathy (PDN).

	Questions	Number, percent correct	Number, percent incorrect
**CLBP knowledge**	CLBP is always related to injury	351, 89.5%	41, 10.5%
	CLBP can be described as aching, burning, stabbing, tingling, dull, or sharp	174, 44.3%	218, 55.7%
	Back pain usually gets worse before it gets better	310, 79.0%	82, 21.0%
	Bed rest is helpful	232, 59.1%	160, 39.1%
	Appropriate dose of morphine for pain is whatever dose relieves the pain as completely as possible	139, 35.4%	253, 64.6%
	Treatment with non-prescription analgesics should be first line	149, 38.0%	243, 62.0%
	Patients should avoid all painful movements	51, 13.0%	341, 87.0%
	Chronic ibuprofen use can worsen blood pressure	339, 86.5%	53, 13.5%
	Mean score (SD), percent and range of questions answered correctly	4.49/8 (± 1.24), 55%, 0–8	
**CHDs knowledge**	Migraine is primarily a disease of the brain, with a well-established neurological basis	119, 30.3%	273, 69.7%
	Muscular factors and stress contribute to chronic tension-type headaches	376, 95.9%	16, 4.1%
	Migraine patients should be asked if headaches inhibit work, school, and household tasks	376, 95.9%	16, 4.1%
	Patients who suffer from severe migraines should try non-prescription medications first, then try prescription medications	166, 42.3%	226, 57.7%
	Headache suffers should guard against medication over-use	371, 94.6%	21, 5.4%
	Prophylactic drug therapy is recommended for chronic headaches	328, 83.7%	64, 16.3%
	Combination analgesics with codeine are reasonable first-line options	210, 53.5%	182, 46.5%
	Triptans should be reserved for patients who have failed at least two other prescription medications	207, 52.8%	185, 47.2%
	Mean score (SD), percent and range of questions answered correctly	5.49/8 (± 1.23), 69%, 0–8	
**PDN knowledge**	PDN is a result of damage to nerves in a hyperglycemic environment	354, 90.3%	38, 9.7%
	PDN results from both peripheral and central sensitization mechanisms	256, 65.3%	136, 34.6%
	All patients with diabetes will develop PDN	353, 90.0%	39, 10.0%
	Optimal glycemic control is the cornerstone of treatment	328, 83.6%	64, 16.4%
	Tramadol is a reasonable initial treatment option	192, 49.0%	200, 51.0%
	Reasonable starting dose for gabapentin in a patient with severe renal failure is 600 mg thrice daily	304, 77.6%	88, 22.4%
	Tricyclic antidepressants are effective	308, 78.6%	84, 21.4%
	Mean score (SD), percent, and range of questions answered correctly	5.34/7 (± 1.21), 76%, 0–7	
	Mean score for all three sections, percent, and range of questions answered correctly	15.28/23 (± 1.41), 66%, 0–23	
	Comparison of mean scores for CLBP, CHD, and PDN	P < 0.001	
**Opioid knowledge**	The number of inadvertent overdoses related to prescription opioids has been increasing in Ontario	376, 95.5%	16, 4.1%
	Patients will not become addicted to their prescription opioid if they have a legitimate pain problem	283, 72.1%	109, 27.9%
	What is the guideline recommended “watchful dose” per day of opioids?	143, 51.8%	133, 48.2%
		Familiar	Not familiar
	Are you familiar with the Canadian guideline for safe and effective opioid use in chronic non-cancer pain?	190, 48.4%	202, 51.6%

Of the questions assessing knowledge of CLBP, 89.5% of responding pharmacists provided a correct answer to the true/false question on the relationship of CLBP to injury, 79.0% on whether back pain gets worse before getting better, 59.1% on the role of bed rest, and 86.5% on the effect of chronic ibuprofen use on blood pressure. Fewer than half of pharmacists provided a correct answer to the true/false question on various descriptions of chronic pain (44.3%), the appropriate dose of morphine for low back pain relief (35.4%), the role of non-prescription analgesics as a first-line treatment option (38.0%), and whether patients should avoid painful movement (13.0%) (Table 2).

Of the questions assessing knowledge of CHDs, 95.9% of responding pharmacists provided a correct answer to the true/false question on factors and stress that contribute to chronic tension-type headaches, 95.9% on whether migraine patients should be asked if their headaches restrict activities, 94.6% on whether headache sufferers should guard against medication overuse, and 83.7% on whether the use of prophylactic drug therapy is recommended for chronic headaches. Respondents scored less well on the true/false question on use of combination analgesics and codeine as a first-line treatment (53.52% provided a correct answer) and appropriate use of serotonin antagonists (i.e., triptans) for headaches (52.8% provided a correct answer). Less than a third of respondents (30.3%) indicated correctly whether migraine is primarily a disease of the brain with a well-established neurological basis, and less than half (42.8%) indicated correctly whether a trial of non-prescription medications should be tried first before taking prescription medications among patients who suffer from severe migraine.

Of the questions assessing knowledge of PDN, 90.6% respondents provided a correct answer to the true/false question whether PDN results from damage to nerves in a hypoglycemic environment and 90.0% provided a correct answer to the question asking if all patients with diabetes will develop PDN. However, about two-thirds (65.3%) provided a correct answer to the question whether PDN results from both peripheral and central sensitization mechanisms. Most (83.6%) correctly answered the question whether optimal glycemic control is the cornerstone of treatment, but just less than half (49.0%) answered the question about tramadol being a reasonable initial treatment option correctly. Slightly more than three-quarters of respondents (77.6%) correctly answered the question whether a reasonable starting dose for gabapentin in a patient with severe renal failure is 600 mg three times a day and 78.6% correctly answered the question whether tricyclic antidepressants are effective (Table 1).

#### Knowledge of opioid management

Slightly less than half (48%) of respondents were familiar with the Canadian guideline for safe and effective opioid use for chronic non-cancer pain. Almost all pharmacists (96%) were aware that inadvertent overdoses related to prescription opioids has been increasing in Ontario, and 72% correctly answered the question whether patients can become addicted to prescription opioids if they have a legitimate pain problem. Only 276 of 392 pharmacists who completed the survey provided responses to the request for the guideline recommended “watchful dose” of opioids per day in mg morphine equivalents. Of these respondents, 52% answered correctly that 200 mg morphine equivalents per day was the recommended dose, and 48% indicated inaccurate doses, ranging from 0 to 2000 mg morphine equivalents per day.

#### Perceptions and attitudes of pharmacists

Slightly greater than half (52%) of responding pharmacists considered complementary therapies (physiotherapy, acupuncture, and exercise) as “very important” to incorporate into treatment of patients with CLBP; however, only 21% and 22% of respondents indicated these complementary therapies as being very important to incorporate into treatment of patients with CHDs and PDN, respectively (p<0.001 across all comparisons). Slightly more than half of respondents indicated that under-dosing with analgesics was moderately important as the reason for ongoing pain, with 52% indicating this for CLBP, 53% for CHDs and 52% for PDN. Almost half of respondents (47%) indicated that opioids were “very important” or “important” to treat CLBP, whereas 23% indicated that opioids were “very important” or “important” to treat CHDs, and 24% indicated that opioids were “very important” or “important” to treat PDN. Few pharmacists expected full improvement from CLBP, CHDs, or PDN (3%, 3% and 1%, respectively); however, 24%, 39%, and 20% expected major improvement and 38%, 48%, and 55% expected moderate improvement in these disorders, respectively.

When asked how they feel when approached by patients with CLBP, CHDs, and PDN, most pharmacists indicated they were comfortable (67%, 62%, and 58%, respectively). In contrast, few pharmacists indicated they felt burdened (7%, 4%, and 4%, respectively).

### Subgroup analyses

#### Differences in responses between male and female respondents

Female respondents were significantly less likely than their male counterparts to answer the following question correctly, “Appropriate dose of morphine for pain is whatever dose relieves the pain as completely as possible,” (56% males vs. 44% females, p<0.05) as related to CLBP (Table 3). Female respondents were significantly more likely than their male counterparts to answer the following question correctly, “Triptans should be reserved for patients who have failed at least two other prescription medications,” (40% males vs. 60% females, OR = 2.0 (95% CI 1.37–3.07), p<0.001). Other responses by gender were not significantly different.

**Table 3:** Proportion of correct responses to knowledge questions on CLBP, CHDs and PDN: Subgroup Analysis

	Questions	Gender			Years of Practice			Hours of Practice/Week			Population Size of Practice		
		Male	Female	P value	≤10	>10	P value	≤24	>24	P value	Rural	Urban	P value
		N = 190 (%)	N = 202 (%)		N = 108 (%)	N = 284 (%)		N = 78 (%)	N = 314 (%)		N = 141 (%)	N = 251 (%)	
**CLBP knowledge**	CLBP is always related to injury	171 (49)	180 (51)	>0.05	94 (27)	257 (73)	>0.05	70 (20)	281 (80)	> 0.05	128 (36)	223 (64)	> 0.05
	CLBP can be described as aching, burning, stabbing, tingling, dull or sharp	82 (47)	92 (53)	>0.05	34 (20)	140 (81)	<0.05	34 (20)	140 (80)	> 0.05	64 (37)	110 (63)	> 0.05
	Back pain usually gets worse before it gets better	147 (47)	163 (53)	>0.05	72 (23)	238 (77)	<0.001	63 (20)	247 (80)	> 0.05	115 (37)	195 (63)	> 0.05
	Bed rest is helpful	111 (48)	121 (52)	>0.05	68 (29)	164 (71)	≥0.05	46 (20)	186 (80)	> 0.05	98 (42)	134 (58)	> 0.05
	Appropriate dose of morphine for pain is whatever dose relieves the pain as completely as possible	78 (56)	61 (44)	<0.05	27 (19)	112 (81)	<0.05	26 (19)	113 (81)	> 0.05	35 (25)	104 (75)	= 0.001
	Treatment with non-prescription analgesics should be first line	78 (52)	71 (48)	>0.05	34 (23)	115 (77)	>0.05	20 (13)	129 (87)	< 0.05	50 (34)	99 (66)	> 0.05
	Patients should avoid all painful movements	26 (51)	25 (49)	>0.05	15 (29)	36 (71)	>0.05	7 (14)	44 (86)	> 0.05	17 (33)	34 (67)	> 0.05
	Chronic ibuprofen use can worsen blood pressure	158 (47)	181 (53)	>0.05	94 (28)	245 (72)	>0.05	65 (19)	274 (81)	> 0.05	127 (37)	212 (63)	> 0.05
**CHDs knowledge**	Migraine is primarily a disease of the brain, with a well-established neurological basis	58 (49)	61 (51)	>0.05	24 (20)	95 (80)	>0.05	24 (20)	95 (80)	> 0.05	35 (29)	84 (71)	> 0.05
	Muscular factors and stress contribute to chronic tension-type headaches	180 (48)	196 (52)	>0.05	99 (26)	277 (74)	<0.05	75 (20)	301 (80)	> 0.05	135 (36)	241 (64)	> 0.05
	Migraine patients should be asked if headaches inhibit work, school and household tasks	182 (48)	194 (52)	>0.05	101 (27)	275 (73)	>0.05	73 (19)	303 (81)	> 0.05	134 (36)	242 (64)	> 0.05
	Patients who suffer from severe migraines should try non-prescription medications first, then try prescription medications	82 (49)	84 (51)	>0.05	49 (30)	117 (70)	>0.05	32 (19)	134 (81)	> 0.05	64 (39)	102 (61)	> 0.05
	Headache suffers should guard against medication over-use	181 (49)	190 (51)	>0.05	98 (26)	273 (74)	<0.05	75 (20)	296 (80)	> 0.05	130 (35)	241 (65)	> 0.05
	Prophylactic drug therapy is recommended for chronic headaches	156 (48)	172 (52)	>0.05	96 (29)	232 (71)	>0.05	58 (18)	270 (82)	< 0.05	115 (35)	213 (65)	> 0.05
	Combination analgesics with codeine are reasonable first-line options	96 (46)	114 (54)	>0.05	60 (29)	150 (71)	>0.05	46 (22)	164 (78)	> 0.05	84 (40)	126 (60)	> 0.05
	Triptans should be reserved for patients who have failed at least two other prescription medications	83 (40)	124 (60)	<0.001	52 (25)	155 (75)	>0.05	41 (20)	166 (80)	> 0.05	80 (39)	127 (61)	> 0.05
**PDN knowledge**	PDN is a result of damage to nerves in a hyperglycemic environment	170 (48)	184 (52)	>0.05	100 (28)	254 (72)	>0.05	70 (20)	284 (80)	> 0.05	122 (34)	232 (66)	> 0.05
	PDN results from both peripheral and central sensitization mechanisms	126 (49)	130 (51)	>0.05	61 (24)	195 (76)	<0.05	55 (21)	201 (79)	> 0.05	84 (33)	172 (67)	> 0.05
	All patients with diabetes will develop PDN	169 (48)	184 (52)	>0.05	98 (28)	255 (72)	>0.05	66 (19)	287 (81)	> 0.05	128 (36)	225 (64)	> 0.05
	Optimal glycemic control is the cornerstone of treatment	163 (50)	165 (50)	>0.05	80 (24)	248 (76)	<0.05	63 (19)	265 (81)	> 0.05	117 (36)	211 (64)	> 0.05
	Tramadol is a reasonable initial treatment option	93 (48)	99 (52)	>0.05	57 (30)	135 (70)	>0.05	34 (18)	158 (82)	> 0.05	73 (38)	119 (62)	> 0.05
	A reasonable starting dose for gabapentin in a patient with severe renal failure is 600mg three times a day	143 (47)	161 (53)	>0.05	81 (27)	223 (73)	>0.05	62 (20)	242 (80)	> 0.05	116 (38)	188 (62)	> 0.05
	Tricyclic antidepressants are effective	146 (47)	162 (53)	>0.05	94 (31)	214 (69)	<0.05	57 (19)	251 (81)	> 0.05	113 (37)	195 (63)	> 0.05

#### Differences in responses based on years of practice

Of the 23 questions on knowledge of different pain conditions, significantly more pharmacists with ten or more years of practice experience answered eight questions correctly (Table 3). Among pharmacists with ten or more years of practice experience, the odds ratios of correctly answering the questions, “Back pain usually gets worse before it gets better,” “Chronic low back pain can be described as aching, burning, stabbing, tingling, dull, or sharp,” “Appropriate dose of morphine for pain is whatever dose relieves the pain as completely as possible,” were 2.56 (95% CI: 1.538–4.276, p<0.001), 2.116 (95% CI: 1.325–3.379, p<0.05), and 2.059 (95% CI: 1.241–3.417; p <0.05), respectively, compared with those with fewer than ten years of experience. Pharmacists who had ten or more years of experience were also more likely to correctly answer questions on factors that contribute to headaches and medication overuse than those with less than ten years of experience (OR = 3.597, 95% CI: 1.305–6.917, p<0.05 and OR = 2.532, 95% CI: 1.043–6.148, p<0.05, respectively). Similarly, pharmacists with ten or more years of experience correctly answered questions related to central and peripheral sensitization in PDN (76% vs. 24%; p<0.05), importance of optimal glycemic control in PDN (76% vs. 24%; p<0.05) and effectiveness of tricyclic antidepressants (69% vs. 31%; p<0.05) than did those with less than ten years of experience.

#### Differences based on population size

Seventy-five percent of pharmacists whose practice location was in an urban area (population >100,000) correctly answered the question, “Appropriate dose of morphine for pain is whatever dose relieves the pain as completely as possible,” whereas among those who practiced in rural locations (population: <10,000–100,000) only 25% did (p<0.001). Other responses by size of city or town in which pharmacists practiced were not significantly significant.

#### Gender, years of practice, population size of location of practice, and opioid management

Differences in the proportions of pharmacists who answered questions correctly on knowledge of opioid management based on gender, years of practice, or population size of practice location were not significant

## Discussion

Respondents had a good understanding of the pathophysiology and pharmacotherapy associated with CLBP, CHDs, and PDN, with an overall score of 66%. Nevertheless, there were statistically significant differences in the overall scores obtained in PDN (76%) as compared to CHDs (69%) and CLBP respectively (76% vs. 69% vs. 55%; p<0.001). The difference in the scores may indicate a greater familiarity with both headache disorders and diabetic neuropathy among pharmacists and a greater need to develop more educational content addressing the pathophysiology and pharmacotherapy of CLBP. For instance, less than 50% of the respondents answered four of eight questions correctly. In the case of the question, “Patients should avoid all painful movements”, only 13% answered correctly and only 38.0% answered the question, “Treatment with non-prescription analgesics should be first line.”

Furthermore, deficiencies were also noted with respect to opioid management. Only about half the respondents were familiar with the 2010 Canadian guideline for safe and effective opioid use for chronic non-cancer pain. While 70% of the 392 who completed the survey attempted to answer the question asking the recommended “watchful dose” per day of opioids in mg morphine equivalents, only half were correct, and incorrect answers ranged widely from 0 to 2,000 mg of morphine equivalents per day. If one were to assume that the remaining 30% did not have adequate confidence to answer this question, only 36% of the pharmacists were aware of the recommended “watchful dose” per day of opioids. The concept of the watchful dose presents a fundamental change in chronic pain treatment with opioids. Previously, it was acceptable to not limit opioid dosing if patients reported continued pain. However, the Canadian guideline includes a recommendation for a watchful dose because “chronic non-cancer pain can be managed effectively in most patients with dosages at or below 200 mg/day of morphine or equivalent” and that “consideration of a higher dosage requires careful reassessment of the pain and of risk for misuse, and frequent monitoring with evidence of improved patient outcomes” [42]. The need for this limit is reinforced by the evidence that the odds of inadvertent overdose increases 3-fold with daily doses above 200 mg of morphine equivalents [17]. It is critical this recommended change is incorporated into frontline practice.

This study’s findings are consistent with previous research indicating that clinicians are generally knowledgeable about opioids but are less adept at handling questions related to their use [21,43–46]. This study’s findings are also consistent with previous research that acknowledges a need for additional education on chronic pain and opioid use [21,47]. Moreover, it demonstrates a need for guideline recommendations to be incorporated into practice. This is a core theme in Canada’s national strategy called “First Do No Harm: Responding to Canada’s Prescription Drug Crisis” [48]. Citing healthcare practitioner knowledge gaps in pain management and addiction, the eight education-related recommendations in the national strategy all relate to the need for increased training for healthcare practitioners.

More than half of the respondents had more than ten years of practice experience—experience that appears to be associated with greater knowledge of CLBP, CHDs and PDN. This finding is consistent with the notion that experience has a substantial direct effect on knowledge in higher complexity employment [49], but it differs from a finding that physicians with more practice experience possessed less accurate factual knowledge [50]. It is possible, however, that respondents in our study were a self-selecting sample who were more interested and engaged in chronic pain care provision than the larger pharmacist population in Ontario.

About 95% of Canada is rural, but depending on the definition used, rural regions comprise 19% to 30% of the country’s population. Of the province-wide professional licensing database, 36% of respondents indicated that they practiced in regions with fewer than 100,000 people. There was largely no difference in knowledge associated with CLBP, CHDs and PDN between urban and rural practicing pharmacists, despite challenges associated with rural clinical practice, such as reduced access to educational opportunities and less peer support [51].

Pharmacists overall were positive about managing these chronic pain conditions, with about two-thirds reporting an expectation of moderate to major improvement. Among the three conditions, complementary therapy was noted to have the strongest role in treatment of CLBP, similar to the proportion who indicated that opioids play a role in treatment. About half of the respondents indicated that under-dosing is a moderately important cause of poor pain control.

### Strengths

This study provides insight into the state of practice of community-based pharmacists with respect to chronic pain care provision in Ontario. Despite a modest response rate, to our knowledge this is the largest survey of pharmacists’ knowledge, attitudes, and perceptions of patients with chronic pain in primary care conducted in Canada. In comparison to Lalonde et al, our study had a larger sample size (392 vs 110 completed surveys), higher male to female ratio (0.6:1 vs. 0.9: 1) and a greater percentage of pharmacists with substantial practice experience (34% vs. 51%)[21].

### Limitations

The low response rate and the possibility that participating clinicians in our study may have been a self-selecting sample because of their experience or interest in chronic pain, may not be representative of Ontario’s pharmacists overall. Furthermore, there is no possibility of assessing the nonresponse bias inherent in a voluntary participation survey. Our survey may have included a higher proportion of pharmacists who manage chronic pain actively, inflating estimates of knowledge scores. Even with pilot testing and question refinement, survey questions may still be ambiguous, result in bias, and, as with all responses that require recall or estimation, responses may have inaccurately reflected practice reality.

## Conclusion

Community pharmacists and pharmacists who are part of family health teams frequently see patients with chronic pain, thus have the opportunity to improve their care. However, this survey demonstrates that pharmacist contributions to the effective and safe treatment of these patients, particularly with opioids, appears to require additional education. Appropriate educational initiatives and pharmacist training to develop skills and provide tools may improve the care of chronic pain patients.

## Supporting Information

S1 File Data of chronic pain knowledge and attitude. (XLSX)

S1 Table Representativeness of population of pharmacists who responded to survey (N = 392) to the Ontario pharmacist population at large. (DOCX)
