# Supplementary material for: Knowledge, Perceptions and Attitudes toward Chronic Pain and Its Management: A Cross-Sectional Survey of Frontline Pharmacists in Ontario, Canada
Source: PLoS One. 2016 Jun 7;11(6):e0157151. doi: 10.1371/journal.pone.0157151 (PMC4896448; doi:10.1371/journal.pone.0157151)
Supplement: S1 Table — (DOCX) [file pone.0157151.s002.docx]

**S1 Table. Representativeness of population of pharmacists who responded to the survey (N = 392) to the Ontario pharmacist population at large**

| **Characteristics** | **No of survey pharmacists in 2014, n=392**  **(percentage of population)** | **No of Ontario pharmacists in 2014, n=13207**  **(percentage of population)** |
| --- | --- | --- |
| **Sex**  Male  Female | 190 (48.5%)  202 (51.5%) | 5591 (42.3%)  7616 (57.7%) |
| **Age**  <30  30-39  40-49  50-59  ≥60 | 21 (5.4%)  72 (18.3%)  114 (29.0%)  113 (28.8%)  72 (18.4%) | 1569 (11.8%)  3455 (26.1%)  3576 (27.0%)  2811 (21.2%)  1796 (13.5%) |
| **Hours of practice**  0-24  25-40  >41 | 76 (19.3%)  230 (58.7%)  86 (21.9%) | 3349 (25.3%)  6500 (49.2%)  2539 (19.2%) |
| **Education**  BSC in Pharmacy  Entry level PharmD  PharmD  MScPhm  Residency trained  Fellowship trained | 370 (94.4%)  4 (1%)  16 (4.1%)  16 (4.1%)  24 (6.1%)  0 | 12468 (94.4%)  - (-) **no data**  391 (2.9%)  233 (1.8%)  -(-)  -(-) |
| **Years of Practice**  0-10  11-20  >20 | 107 (27.3%)  84 (21.4%)  201 (51.3%) | 3662 (27.7%)  3319 (25.1%)  6188 (46.8%) |

The table above demonstrates that data from the Chronic Pain survey in 2014 was obtained from pharmacists that are representative of the Provincial level data of practicing pharmacists in Ontario in 2014. The comparison shows that sex ratios, hours of practice, education levels and years of practice were similar between groups. The more senior age groups were slightly more represented than the younger age groups in the survey respondents, as compared with all Ontario pharmacists.

Regional pharmacists’ data, 2014 was obtained from the Canadian Institute of Health Information

**Reference:**

Canadian Institute for Health Information. Pharmacists, 2014. November 26, 2015. Available from [https://secure.cihi.ca/estore/productFamily.htm?locale=en&pf=PFC3041&lang=en](https://secure.cihi.ca/estore/productFamily.htm?locale=en&pf=PFC3041&lang=en). Accessed April 5, 2016.
